# A case report of confusing meningoencephalocele

**DOI:** 10.1002/ccr3.3470

**Published:** 2020-12-04

**Authors:** Marika Perrotta, Giorgia Gasparroni, Valentina Chiavaroli, Luca Massimi, Rita Cognigni, Laura Sabatini, Teresa Topazio, Silvia Carinci, Marianna Sebastiani, Angelika Mohn, Susanna Di Valerio

**Affiliations:** ^1^ Department of Pediatrics Gabriele d'Annunzio University of Chieti and Pescara Chieti Italy; ^2^ Pescara Public Hospital Neonatal Intensive Care Unit Pescara Italy; ^3^ The University of Auckland Liggins Institute Auckland New Zealand; ^4^ Policlinico Universitario Agostino Gemelli Neurochirurgia Infantile Roma Italy; ^5^ Università Cattolica del Sacro Cuore Facoltà di Medicina e Chirurgia Istituto di Neurochirurgia Roma Italy

**Keywords:** encephaloceles, meningoencephalocele, newborn, scalp swelling

## Abstract

The clinical approach plays a pivotal role in neonates with evidence of a skull mass, together with the need of monitoring unclear cases. Indeed, apparently transient alterations of the skull may be neural tube defects, which need prompt treatment.

## Graphical abstract

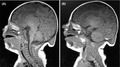

## 1 INTRODUCTION

A smooth mass overlying the scalp was observed in a newborn. Although initially labeled as a cephalohematoma, the clinical features and persistence over the weeks were suggestive of a neural tube defect. An occipital meningocele was detected by magnetic resonance imaging. The final diagnosis of meningoencephalocele was made during surgery.

In the human embryo, neural tube closure takes place during the process of primary neurulation. This complex phenomenon begins at the level of the craniocervical junction and progresses rostrally and caudally in a zipper‐like manner.^1^ Any defects of this process may lead to defective development of the structures along the craniospinal axis.^1^, ^2^ Worldwide, neural tube defects occur in approximately 1/1000 pregnancies, with a geographical variation of the disease prevalence.^3^ Indeed, in the United States and many European countries, the prevalence of neural tube defects is 0.5‐0.8/1000 births,^4^ while the prevalence is more than 20 times higher in some regions of China.^5^ The diagnosis is usually straightforward at birth (or made during pregnancy, in case of open and/or massive defects). However, in some instances, the defect may remain undiagnosed or misdiagnosed.

Here, we report the case report of a female newborn with a meningoencephalocele, which was diagnosed after birth and whose diagnosis was initially controversial.

## 2 CASE HISTORY/EXAMINATION

This female newborn was delivered at 38 weeks’ gestational age by vaginal delivery, which progressed without requiring medical interventions. At delivery, the Apgar score was 7 at 1 minute and 9 at 5 minutes of life. Birthweight was 2540 gr (−1.28 SDS, 10th percentile). The girl was the first offspring of unrelated healthy parents. Family history was negative for congenital malformations and disorders. Mother had gestational hypothyroidism from early pregnancy, which was treated with levothyroxine; pregnancy was otherwise uneventful. Also, prenatal vitamins including the recommended 400 micrograms of folic acid daily were regularly taken in the first trimester of pregnancy (ie, folic acid supplement was started approximately 5 months before conception).

At the initial full clinical examination performed soon after birth, the newborn's general appearance and clinical conditions were normal. No congenital anomalies were detected. However, the skull examination showed a well‐circumscribed mass overlying the scalp in the occipital region. Clinical examination of the mass revealed a circular shape with regular margins, overlying skin not discolored and a size of approximately 3 × 3 cm. Palpation detected a smooth surface and a soft consistency of the mass, which was neither fluctuating nor reducible. No restricted neck extension or neurological deficit was noted.

### 2.1 Differential diagnosis, investigations, and treatment

Differential diagnosis included caput succedaneum, although this hypothesis was improbable as the swelling did not cross sutures and was well‐demarcated. Also, bruising, ecchymosis, and/or petechiae were absent. A subgaleal hemorrhage was excluded because this life‐threatening condition is not limited by cranial suture lines and appears palpably firm to fluctuant. Lastly, a neural tube defect (eg, encephalocele) was not suspected as, on the first impression, the skull swelling was interpreted as cephalohematoma, secondary to a possible unnoticed mechanical birth‐related trauma.

At 2 days of life, phototherapy was required for 24 hours due to hyperbilirubinemia, which was considered a side effect of the cephalohematoma; afterward, bilirubin levels remained in the normal range.

Over the days spent at the hospital, the size of the occipital tumefaction remained identical. This finding was unexpected as a cephalohematoma may increase in size for several days. Transillumination of the neonatal skull was not evaluated while a brain ultrasound was performed, which showed no abnormalities. The newborn was discharged at 5 days of life in good clinical conditions with a follow‐up program of the supposed cephalohematoma, which was expected to resolve spontaneously over the following weeks.

At the 15‐day and 1‐month follow‐up visits, the occipital tumefaction was still found to persist. The newborn was otherwise in good clinical conditions, with regular body growth and development. The clinical finding of an unchanged mass, in both size and consistency, and its peculiar localization in the occipital region, leads to consider an alternative diagnosis, and a neural tube defect was suspected. Thus, magnetic resonance imaging of the brain was performed. Magnetic resonance imaging scans were performed on a Philips 1.5‐T Scanner (Philips Medical Systems Nederland). The imaging protocol included the sequences: T1‐weighted, T2‐weighted, fluid‐attenuated inversion recovery, diffusion‐weighted imaging, and susceptibility‐weighted imaging.

**FIGURE 1:**
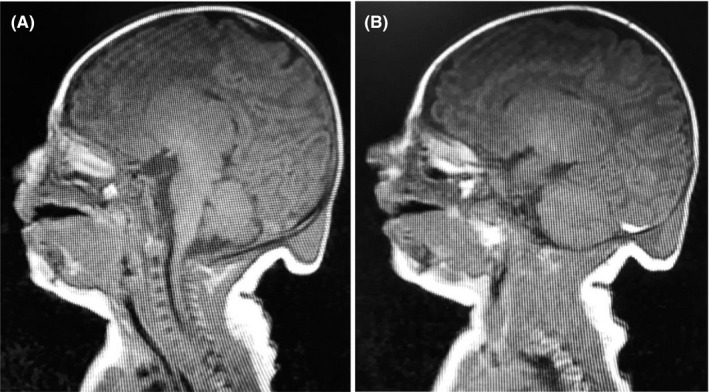
Sagittal magnetic resonance images showing (A) a small hyperintense collection in the left median‐paramedian occipital site, located under the skull; and (B) a fluid oval collection in the retronuchal site, with an inhomogeneous signal and a small fluid level in its context

Magnetic resonance imaging showed a small hyperintense collection in the left median‐paramedian occipital site, located under the skull (maximum thickness: 6 mm). Moreover, a fluid oval collection was observed in the retronuchal site (maximum axial diameter: 3 cm), with an inhomogeneous signal and a small fluid level in its context (Figure 1A,B). An occipital meningocele was diagnosed and surgical repair was electively performed, which was uneventful. During surgery, dysplastic brain tissue was detected in the mass, leading to a final diagnosis of meningoencephalocele.

### 2.2 Outcome and follow‐up

The excised part was sent for histopathological examination, which confirmed the diagnosis of meningoencephalocele. The postoperative course was unremarkable and the patient was discharged in good clinical conditions, with a follow‐up program. Over the following months, the patient showed regular physical growth as well as normal motor and cognitive development.

## 3 DISCUSSION

Neural tube defects are included among the most common, severe congenital anomalies, together with congenital heart defects and abnormalities of urinary system.^6^ Several central nervous system anomalies belong to the neural tube defect spectrum, such as encephaloceles, anencephaly, and spina bifida.

Encephaloceles are associated with defects in the skull vault bones.^7^ Namely, a partial defect may occur in bone fusion, leading to the herniation of meninges (cranial meningocele), brain tissue (meningoencephalocele), or brain plus spinal cord tissue (encephalomyelocele). These structural abnormalities may occur everywhere on the skull, although the occipital region is most commonly involved (70%‐90% of cases).^8^ The prevalence of encephaloceles is 0.8‐4/10.000 live births.^7^ An increased prevalence has been reported among girls and in those with a birthweight <2500 gr.^9^

Meningoencephalocele is defined as the herniation of neural elements (usually a degenerative cerebral cortex), cerebrospinal fluid, and meninges through a congenital defect in the cranium. When meningoencephalocele is located in the occipital region, herniation of meninges, occipital lobes, and/or ventricles are common. Cerebellum, brainstem, or, rarely, torcula can also be detected.^10^

These complex anomalies of the central nervous system are multifactorial, involving both genetic and environmental causes.^11^ The latter comprises several factors, including maternal exposure to environmental hazards (eg, occupational pesticide exposure), comorbidities (eg, obesity and diabetes), infections (eg, cytomegalovirus and toxoplasma),^8^ and nutritional factors. Of note, an adequate maternal intake of folic acid before and during pregnancy has been shown to significantly decrease the risk of developing neural tube defects (up to 70%).^11^ In this report, it has to be acknowledged that the mother started folic acid supplement before conception, continuing it during the first trimester of pregnancy. Nevertheless, variants of folate‐related genes may alter folate metabolism, thus raising the risk of having a baby with a neural tube defect.^11^

Hypothyroidism is considered as a metabolic condition that can cause teratogenic effects and hazard the growing embryo, also affecting the skeletal tissue.^12^ Here, the mother suffered from gestational hypothyroidism from early pregnancy. However, levothyroxine therapy was promptly started, thus allowing to reach biochemical euthyroidism that was maintained throughout pregnancy.

In most cases, antenatal fetal ultrasound, possibly followed by fetal magnetic resonance imaging, allows to detect skull vault bone defects and to visualize the herniated structure contents.^8^, ^13^ The diagnostic protocol in those newborns with a postnatal evidence of scalp abnormalities includes a careful examination of anatomical location (eg, anterior or posterior), size (small swelling may be at first undiagnosed or misdiagnosed), shape, margins, overlying skin, consistency, and translucence.^13^ A skin‐covered swelling of the scalp, which is placed close to the midline and may appear translucent, is suggestive of a neural tube defect.^8^ It is also mandatory to check whether there is evidence of further cranial and extracranial defects (eg, hydrocephalus and facial abnormalities) ^13^ as well as neurological deficits.^14^ Indeed, up to 60% of newborns with encephalocele suffer from concurrent cranial and/or extracranial defects.^13^, ^14^ Magnetic resonance imaging of the brain represents the best diagnostic tool in infants suspected to have an encephalocele. Indeed, it allows to identify the skull defect and the sac contents as well as any further abnormalities.^8^

Elective surgery is the treatment of choice of meningoencephaloceles, and the characteristics of the defect must be taken into account.^15^ Indeed, surgery of large meningoencephaloceles, where functional nervous tissue is contained, can be complex. Timing of surgical intervention and the outcome of patients with occipital meningoencephaloceles depend on several factors (eg, site, size, amount of brain herniated).^16^ In this report, the surgical repair was electively performed and postoperative recovery was uneventful. Besides, follow‐up assessment revealed a normal motor and cognitive development.

In conclusion, this case report emphasizes the importance of the clinical assessment in newborns with evidence of scalp abnormalities and the need of performing a close follow‐up in clinically unclear cases. Although relatively uncommon, newborns with a supposed transitory swelling of the scalp may instead suffer from neural tube defects, such as meningoencephalocele, which require to be promptly diagnosed and treated.

## CONFLICT OF INTEREST

None declared.

## AUTHOR CONTRIBUTIONS

MP, GG, and VC: wrote the manuscript. RC, SDV, and MS: examined the patient. LM: made the final diagnosis. TT and LS: were involved in the literature search and drafting of the paper. SC and AM: coordinated and approved the final version of the manuscript. The content has not been published or submitted for publication elsewhere.

## ETHICAL APPROVAL

Verbal and written consent was obtained from the parents regarding the publication of the case and images. This report does not contain any personal information that could lead to the identification of the patient.

## Data Availability

Data sharing not applicable—no new data generated.
